# High rates of maternal depression amongst Syrian refugees in Lebanon - a pilot study

**DOI:** 10.1038/s41598-019-48247-5

**Published:** 2019-08-14

**Authors:** Kerrie Stevenson, Reina Alameddine, Ghaith Rukbi, Mario Chahrouri, Jinan Usta, Bassem Saab, Phillip Bennett, Vivette Glover, Rebecca M. Reynolds

**Affiliations:** 1 Institute of Reproductive and Developmental Biology, Imperial College London, Hammersmith Campus, Du Cane Road, London, W12 ONN UK; 2 American University of Beirut Medical Center, Department of Family Medicine, Riad El Solh, Beirut, Lebanon; 3 University of Edinburgh, Centre for Cardiovascular Science, Queen’s Medical Research Centre, 47 Little France Crescent, Edinburgh, Scotland EH16 4TJ UK

**Keywords:** Depression, Risk factors, Epidemiology

## Abstract

This pilot study compares symptoms of depression and risk factors amongst Syrian refugees and low-income Lebanese mothers accessing a primary care centre in Beirut between January and June 2018. Women who gave birth in the previous two years or who were currently pregnant were included in the study. Depressive symptoms were assessed using the Arabic Edinburgh Postnatal Depression Scale (EPDS). Correlations between EPDS score and sociodemographic and mental health variables were analysed using Pearson’s coefficient and ANOVA. 35 Syrian and 25 Lebanese women were recruited, 15 of whom were pregnant. EPDS scores were high in the whole group (mean 16.12 (SD 7.72), n = 60). Scores were higher amongst Syrian refugees than Lebanese mothers (17.77, SD 7.66 vs, 13.80, SD 7.34, *p* < 0.05). Illegal residence (*p* < 0.001), domestic violence (*p* < 0.05) and a history of mental illness (p < 0.01) were associated with higher scores. This pilot study demonstrates high rates of symptoms of depression amongst mothers in this population. Symptoms were particularly prevalent amongst Syrian refugees; three-quarters were ‘probably depressed’ and would warrant psychiatric assessment. This highlights the importance of improved mental healthcare for refugee mothers, the importance of addressing the social determinants of maternal mental health and further research into the effects of depression on these women and their children.

## Introduction

Since 2012 the Syrian conflict has resulted in the displacement of 5.5 million people, approximately one million of whom are registered in Lebanon^1,2^. Together with a large number of unregistered Syrians and refugees from the surrounding region, approximately 25% of the Lebanese population is a refugee, meaning it hosts the largest number of refugees per capita of any other country^3^. In this paper the term refugee is used to refer to migrants with both official and unofficial asylum status.

There are known inadequacies in gynaecological, reproductive, obstetric and mental healthcare for female refugees in Lebanon^1,4^. An estimated 50,000 Syrian refugees gave birth in Lebanon in 2017^5^. Despite efforts from the UNHCR to improve pregnancy care, only 73% of refugees access any form of antenatal care and just 41% receive a minimum of four antenatal visits as recommended in the World Health Organisation’s (WHO) Millennium Development Goals (MDG)^6^. These women may be at particular risk of developing mental illness^7,8^. Prospective studies have demonstrated that perinatal maternal mental illness increases the risk of adverse outcomes for the child including impaired cognitive development and attention deficit hyperactivity disorder^9^. Despite the scale of the Syrian refugee crisis, a very small number of studies investigating maternal depression amongst Syrian refugee mothers in host countries exist^8,10^. The prevalence of perinatal depression amongst Syrian refugee mothers in Lebanon is unknown.

The primary aim of this pilot study was to estimate the prevalence of depressive symptoms amongst Syrian refugee mothers accessing a primary care centre in Beirut. The secondary aim was to identify risk factors for maternal depression by examining the associations with sociodemographic factors and a history of mental illness.

## Methods

### Participants

This study was conducted in a primary care centre located in the West Sabra district of Beirut, Lebanon, which predominantly serves individuals living in one of Beirut’s illegal urban settlements. The study incorporates very-low income Lebanese women and Syrian refugee mothers who settled there since the onset of the Syrian war. Women who had given birth in the previous two years or who were currently pregnant were eligible for inclusion. This study is part of a larger study investigating maternal health and wellbeing in this population of women. Recruitment and data collection took place between January and June 2018. Women were recruited by non-medical staff enabling women to elect or decline to participate confidentially without the risk of pressure from their clinicians. Women were excluded if they were under eighteen years of age, were unable to provide informed consent or if they were not a Syrian refugee or a Lebanese woman. Women with limited literacy were read a pre-prepared Arabic explanation script. Informed consent was obtained, and each participant given a unique anonymous subject number. Women who elected to participate attended the clinic at a later date to meet with a medical resident who may have been involved in their medical care in the past. This did not impact their recruitment and involvement in the study.

### Compliance with ethical standards

This study was approved by the Institutional Review Board, American University of Beirut, Lebanon and endorsed by the Research Ethics Committee, University of Edinburgh, Scotland. All procedures involving human participants were conducted in accordance with the ethical standards of the institutional research committee and with the 1964 Helsinki declaration and its later amendments or comparable ethical standards. Informed consent was obtained from all individual participants included in the study. With consent, women were offered additional psychological support if they experienced increased psychological distress due to involvement in the study or if the medical residents felt this was warranted.

### Assessing risk factors for maternal depression

Sociodemographic and mental health-related data was collected via a peer-reviewed and piloted questionnaire, which was created in conjunction with the research team, clinicians working in the primary care centre and a focus group of women involved in the study. Basic sociodemographic and obstetric history data was collected: maternal age, age of marriage, illegal residence status and parity. Variables known to be associated with increased risk of maternal depression including history of mental illness, illegal residence and exposure to domestic violence were assessed^9,11^. Owing to high rates of illiteracy all questionnaire data was collected verbally in Arabic, and researchers conducting interviews attended a three-hour training session in advance of data collection. Following consultation, it was decided that all risk factors for depressive symptoms would be assessed via a single closed question in order to minimise psychological distress to the women, such as, ‘What is your marital status?’, ‘Have you been diagnosed with any mental health conditions?’, ‘Have you experienced domestic violence?’. Definitions were not provided to the women and researchers were asked not to prompt women for answers. We were unable to verify any of the diagnoses of mental illness owing to lack of access to health records, and we did not ask women to specify if their diagnosis had been made by a medical professional. At the time of study design we were unable to find any validated approaches to collecting data related to depressive symptoms in this population of women.

### Maternal depression screening instrument

Rates of maternal depression were assessed using a validated Arabic version of the Edinburgh Postnatal Depression Scale (EPDS)^12^. The EPDS is commonly used to screen for symptoms of depression, including anxiety, feelings of guilt, and dysphoria, during and following pregnancy. This ten-item self-reported questionnaire assesses the frequency of depressive symptoms over the preceding seven days and scored using a four-point scale (0–3). A higher score indicates a higher degree of depressive symptoms. As recommended by the instrument authors, a score of thirteen or more was regarded as ‘*probable depression*’, indicating the need for a mental health referral. A score of ten to twelve suggests ‘*possible depression’*.

### Statistical analysis

Data analysis was carried out using IBM SPSS statistical software, version 24^13^. All data were normally distributed. Analyses compared differences in sociodemographic and mental health data for Syrian and Lebanese women. Continuous variables were compared using an independent t-test and mean and standard deviation (SD) are presented. Categorical variables were compared using a chi-squared test. Correlations between, or differences in, EPDS score and the variables in Table 1 were analysed using Pearson’s coefficient (*r*) and ANOVA, as appropriate. Regression analysis was used to identify variables in Table 1 independently associated with mean EPDS score. Data available upon request from the authors.

**Table 1:** Comparison of sociodemographic and mental health variables between all mothers, and then compared between Syrian and Lebanese mothers.

	All Mothers n = 60		*Syrian Refugee Mothers n* = 35		*Lebanese Mothers n* = 25		*p*-value
	n (%)		n (%)		n (%)		
**Age**, **years**							
**mean (SD)**		28.02 (6.92)		28.09 (6.40)^†^		27.92 (7.75)^†^	0.927^†^
*18–24*	21 (35.0%)		12 (34.3%)		9 (36.0%)		
*25–34*	25 (41.7%)		16 (45.7%)		9 (36.0%)		
*35–45*	13 (21.7%)		7 (20.0%)		6 (24.0%)		
*Unknown*	1 (1.7%)		0 (0.0%)		1 (4.0%)		
**Child Marriage: aged <18 years**							
**mean** (**SD**)		18.33 (4.09)		19.41 (4.51)^†^		16.79 (2.84)^†^	<0.05*^†^
*12–17*	27 (45.0%)		13 (37.1%)		14 (56.0%)		
*18+*	31 (51.7%)		21 (60.0%)		10 (40.0%)		
*Unknown*	2 (3.3%)		1 (2.9%)		1 (4.0%)		
**Illegal residence in Lebanon**							
*Yes*	10 (16.7%)		9 (25.7%)		1 (4.0%)		0.05*^‡^
*No*	50 (83.3%)		26 (74.3%)		24 (96.0%)		
**Parity**							
**mean (SD)**		3.13 (2.10)		3.09 (2.27)^†^		3.20 (1.87)^†^	0.337^†^
*Nulliparous*	2 (3.3%)		2 (5.7%)		0 (0.0%)		
*1*	13 (21.7%)		9 (25.7%)		4 (16.0%)		
*2*	14 (23.3%)		6 (17.1%)		8 (32.0%)		
*3*	12 (20.0%)		8 (22.9%)		4 (16.0%)		
*4+*	19 (31.7%)		10 (28.6%)		9 (36.0%)		
**Exposure to any form of domestic violence**							
*Yes*	19 (31.7%)		12 (34.3%)		7 (28.0%)		0.606^‡^
*No*	41 (68.3%)		23 (65.7%)		18 (72.0%)		
**History of mental illness**							
*Yes*	16 (26.7%)		8 (22.9%)		8 (32.0%)		0.430^‡^
*No*	44 (73.3%)		27 (77.1%)		17 (68.0%)		

^*^p < 0.05.

^†^Independent *t*-test.

^‡^Chi-squared (χ^2^) test.

SD – Standard Deviation.

## Results

### Sociodemographic and maternal mental health history comparisons

35 Syrian and 25 Lebanese women were recruited, 15 of whom were pregnant (9 Syrian and 6 Lebanese). The sociodemographic and mental illness history for all women (Table 1), was then compared between Syrian and Lebanese women. The youngest woman was 18 years, the oldest 45 years. All women were married apart from 4 Syrian women who were divorced. Rates of exposure to any type of domestic violence were 31.7% (n = 19). Rates of child marriage (<18 years) were significantly higher amongst Lebanese than Syrian women (56.0% (n = 14) vs. 31.7% (n = 13), p < 0.05). Illegal residence rates were significantly higher amongst Syrian refugees compared with Lebanese women (25.7% (n = 9) vs. 4.0% (n = 1), p < 0.05). 26.7% (n = 16) of all women had a history of mental illness.

### Rates of maternal symptoms of depression

**Figure 1:**
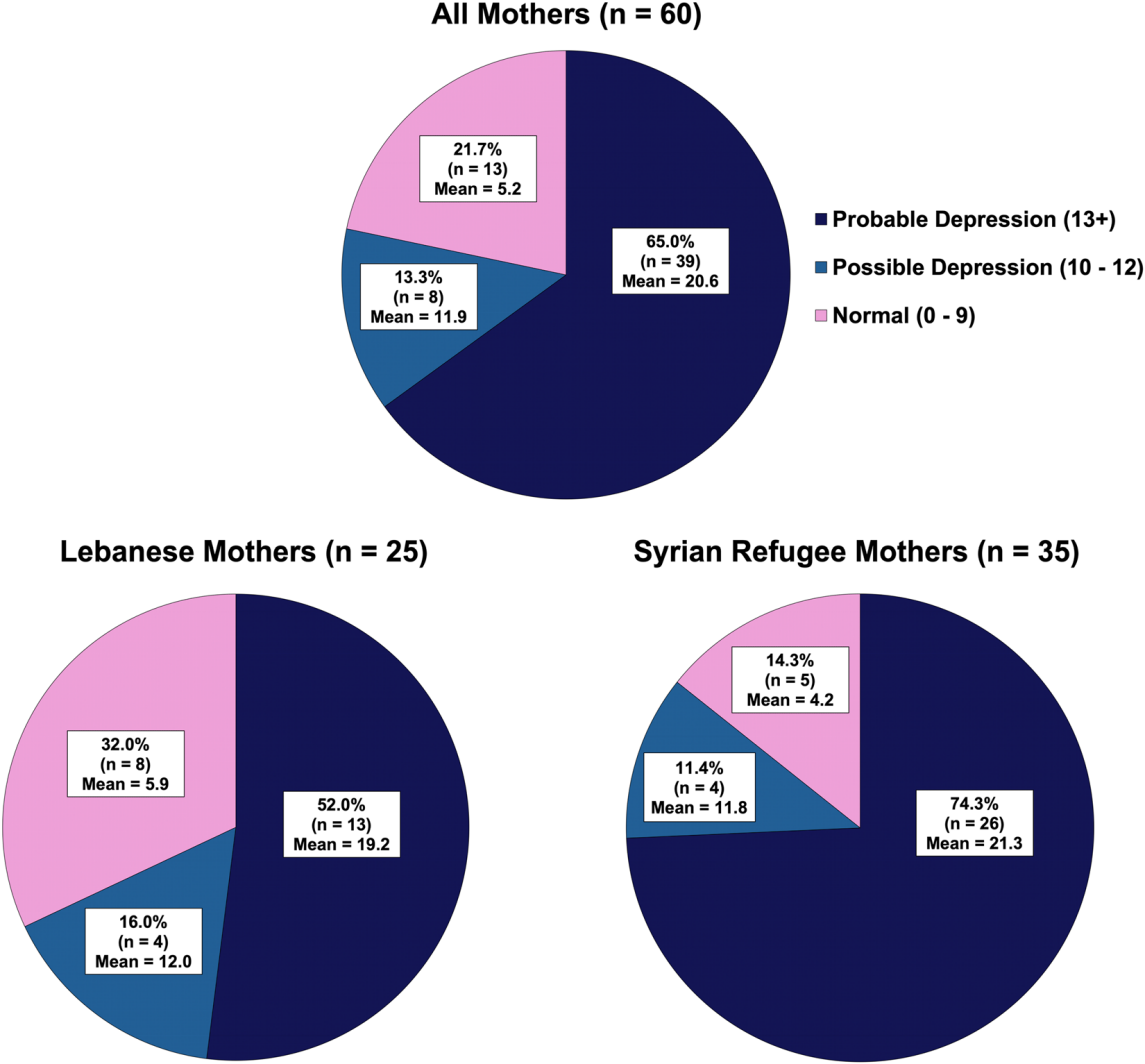
Edinburgh Postnatal Depression Scores according to category of severity of symptoms in all mothers, Lebanese mothers and Syrian refugee mothers. Few symptoms of depression and likely not depressed (EPDS score 0–9), possible depression (EPDS score 10–12), probable depression (EPDS 13+).

The EPDS scores were very high in the whole group (mean 16.12, SD 7.72; n = 60), with 78% (n = 47) of mothers possibly or probably depressed (13.3%, n = 8 and 65.0%, n = 39, respectively) (Fig. 1). Scores were significantly higher amongst Syrian mothers than Lebanese (mean 17.77, SD 7.66 vs, mean 13.80, SD 7.34, *p* < 0.05). There was no difference in mean scores between those who were pregnant and those who were not (data not shown).

### Link between edinburgh postnatal depression scores and sociodemographic variables

Of the variables in Table 1, for all mothers there was a significant correlation between a higher EPDS score with younger age of marriage (*r* = −0.305, *p* < 0.05) and higher mean (SD) scores were associated with illegal residence (Yes: 22.60 (4.79) vs. No: 14.82 (7.57), *p* < 0.01), increased exposure to domestic violence (Yes: 19.68 (6.80) vs. No: 14.46 (7.63), *p* < 0.05) and a history of mental illness (Yes: 20.88 (7.46) vs. No: 14.39 (7.13), *p* < 0.01). In the Syrian subgroup there was a significant correlation between a younger age of marriage and higher EPDS score (*r* = −0.415, *p* < 0.05). Higher mean scores were also associated with illegal residence (Yes: 22.44 (5.05) vs. No: 16.15 (7.81), *p* < 0.05), exposure to domestic violence (Yes: 22.08 (5.14) vs. No: 15.52 (7.87), *p* < 0.05) and a history of mental illness (Yes: 23.00 (5.76) vs. No: 16.22 (7.54), *p* < 0.05). In the Lebanese subgroup a higher EPDS score was associated with a history of mental illness (Yes: 18.75 (8.71) vs. No: 11.47 (5.43), p < 0.05).

Regression analysis showed that within the whole group, higher mean EPDS scores were associated with mental illness (standardised regression coefficient *β* = 0.331, *p* < 0.01), exposure to domestic violence (*β* = 0.255, p < 0.05), and illegal residence (*β* = 0.379, p < 0.001), but not with maternal age at assessment (*β* = −0.154, p = 0.195) (overall model, R square 0.359, p < 0.001).

## Discussion

This pilot study demonstrates very high rates of maternal depressive symptoms amongst both Syrian and Lebanese mothers attending this primary care centre in an informal urban settlement in Beirut. The associations and various risk factors are broadly the same as those reported in similar populations. Symptoms were particularly prevalent amongst Syrian refugees with results demonstrating that three-quarters were ‘probably depressed’ (EPDS score of 13 or more). This contrasts with a smaller Canadian study incorporating 12 resettled Syrian refugee mothers which demonstrated that 25% of the women were ‘probably depressed’^8^. A study involving 365 post-partum Syrian refugee mothers in Jordan found that 50% of women were ‘probably depressed’^10^. In this pilot study, risk factors associated with increased rates of maternal depressive symptoms were illegal residence, a history of mental illness and exposure to domestic violence. Maternal age at assessment was not associated with more depressive symptoms. Although being a younger mother may not cause depression, it could be correlated with risk factors such as poverty or lack of education which could in turn predict depression. The Jordanian study found a correlation between higher EPDS score and low social support, low income and recent arrival in Jordan^10^. The Canadian study assessed risk factors for maternal depression through focus group discussions. Syrian mothers expressed excess stress as a result of refugee status in a Western country, a lack of social support and lack of access to mental health services, but no comparison was made with EPDS score^8^. A systematic review of the prevalence and determinants of perinatal mental health disorders in low- and lower-middle-income countries found that lower maternal age, intimate partner violence and a history of mental illness were associated with higher rates of any of the common perinatal mental disorders. The review did not investigate risk factors for maternal depression alone^11^.

These results demonstrate the importance of addressing the social determinants of mental illness as well as the need for improved access to mental healthcare. Illegal residence was a significant predictor of maternal depressive symptoms amongst all the mothers. The provision of legal housing to Syrian refugee and Lebanese women living in this informal settlement must be a priority for the local government as well as international aid agencies operating in the region^14^. The results highlight the need for interventions to prevent child marriage amongst the families in the study, particularly Lebanese girls who may be particularly susceptible^1,4^. There is a pressing need for parliamentary approval of a draft law introduced in 2017 to set the minimum age for marriage at 18 throughout Lebanon^15,16^. Domestic violence was also significantly associated with depressive symptoms in this study. Further studies are needed gain comprehensive prevalence estimates of domestic violence amongst these women, which should be used to help shape primary and secondary prevention programmes. Failure to address the social determinants of maternal depression in this population may not only increase the prevalence of symptoms, but may also worsen symptoms and impact the success of treatment.

Our results also highlight the need for improved access to mental healthcare amongst Syrian refugee and very-low income Lebanese mothers in Beirut, who may have high rates of depressive symptoms. Despite coordinated efforts by the UNHCR and the Ministry of Public Health to improve mental healthcare services in official Lebanese primary healthcare centres, universal coverage is lacking^17^. In the charity-run primary care centre in which this study was based, one psychologist is available fourteen hours per week for approximately 3,000 patients. No specialist psychiatric care is available in the centre, but women can be referred to external specialist services.

The study is limited by its small sample size from a single urban population of mothers aged 18 years or more, and who are attending a primary care centre in Beirut. As a result, it may not be applicable to other Syrian mothers such as those not accessing healthcare or those in rural areas. In order to better assess the prevalence of depressive symptoms amongst Syrian refugee mothers in Lebanon, a larger sample size and a sample representative of the wider population is needed. Sociodemographic and risk factor data was collected by non-blinded medical residents using self-reported data from women which may be subject to both observer and recall bias. Risk factors for maternal depressive symptoms including a history of mental health problems and exposure to domestic violence were assessed via closed questioning without a definition or prompting. Thus, interpretations may differ in regard to, for example, what constitutes domestic violence. It is also possible that current depressive symptoms may have influenced retrospective reporting of risk factors.

The Arabic EPDS screening tool was validated in the United Arab Emirates (UAE), and was deemed to have good sensitivity and specificity (sensitivity: 73–90%; specificity: 84–90%)^12^. However, the positive predictive value (PPV) was relatively low (PPV: 44–50%), meaning a significant number of false positives can be expected. Whilst the EPDS has been validated in the region, it has not been validated in a refugee population and it’s not clear how this might affect its validity. For example, EPDS questions may be confounded by the refugee experience and may no longer be reliable indicators of clinical depression. However, it is challenging to distinguish confounding factors from genuinely increased depression in this population. In addition, our estimates of prevalence are based on the use of a screening tool alone without the use of a clinical interview, which increases the risk of false positives.

This pilot study is limited in its ability to make an estimate of maternal depressive symptoms amongst the wider Syrian refugee population in Lebanon. However, it can be used to highlight the need for further studies to determine the prevalence of maternal depression and risk factors in the maternal Syrian refugee population in Lebanon. In particular, it emphasises the need for a larger representative sample of mothers and the importance of using clinical interviews as well as screening tools in diagnosing depression. It also highlights the need for validation of the EPDS in the Syrian refugee population and the importance of considering how risk factors for depression are assessed.

To our knowledge this is the first study to investigate rates of depressive symptoms and risk factors amongst Syrian refugee mothers in Lebanon. Despite its limitations and small sample size, the results are consistent with research in similar refugee populations. The study demonstrates an urgent need for a more comprehensive assessment of the prevalence maternal depression and risk factors amongst Syrian refugees in Lebanon. It also stresses the need for action to address the social determinants of poor maternal mental health in this population of mothers, together with the impact on their children, who may well have raised levels of emotional, behavioural and cognitive problems as a result of maternal refugee status.

### Compliance with ethical standards

This research involved human participants. All procedures performed in studies involving human participants were in accordance with the ethical standards of the institutional research committee and with the 1964 Helsinki declaration and its later amendments or comparable ethical standards. Informed consent was obtained from all individual participants included in the study.
